# The mitochondria-related gene risk mode revealed p66Shc as a prognostic mitochondria-related gene of glioblastoma

**DOI:** 10.1038/s41598-024-62083-2

**Published:** 2024-05-19

**Authors:** Gang Peng, Yabo Feng, Xiangyu Wang, Weicheng Huang, Yang Li

**Affiliations:** 1 Department of Phamacology, Xiangya Hospital, Central South University, 87 Xiangya Road, Changsha, 410008 Hunan People’s Republic of China; 2 PET-CT Center, Chenzhou First People’s Hospital, Chenzhou, 423000 Hunan People’s Republic of China; 3 Department of Neurosurgery, Xiangya Hospital, Central South University, 87 Xiangya Road, Changsha, 410008 Hunan People’s Republic of China

**Keywords:** Glioblastoma, p66Shc, Mitochondria, Immune infiltration, ROS, CNS cancer, Computational biology and bioinformatics, Computational models

## Abstract

Numerous studies have highlighted the pivotal role of mitochondria-related genes (MRGs) in the initiation and progression of glioblastoma (GBM). However, the specific contributions of MRGs coding proteins to GBM pathology remain incompletely elucidated. The identification of prognostic MRGs in GBM holds promise for the development of personalized targeted therapies and the enhancement of patient prognosis. We combined differential expression with univariate Cox regression analysis to screen prognosis-associated MRGs in GBM. Based on the nine MRGs, the hazard ratio model was conducted using a multivariate Cox regression algorithm. SHC-related survival, pathway, and immune analyses in GBM cohorts were obtained from the Biomarker Exploration of the Solid Tumor database. The proliferation and migration of U87 cells were measured by CCK-8 and transwell assay. Apoptosis in U87 cells was evaluated using flow cytometry. Confocal microscopy was employed to measure mitochondrial reactive oxygen species (ROS) levels and morphology. The expression levels of SHC1 and other relevant proteins were examined via western blotting. We screened 15 prognosis-associated MRGs and constructed a 9 MRGs-based model. Validation of the model's risk score confirmed its efficacy in predicting the prognosis of patients with GBM. Furthermore, analysis revealed that SHC1, a constituent MRG of the prognostic model, was upregulated and implicated in the progression, migration, and immune infiltration of GBM. In vitro experiments elucidated that p66Shc, the longest isoform of SHC1, modulates mitochondrial ROS production and morphology, consequently promoting the proliferation and migration of U87 cells. The 9 MRGs-based prognostic model could predict the prognosis of GBM. SHC1 was upregulated and correlated with the prognosis of patients by involvement in immune infiltration. Furthermore, in vitro experiments demonstrated that p66Shc promotes U87 cell proliferation and migration by mediating mitochondrial ROS production. Thus, p66Shc may serve as a promising biomarker and therapeutic target for GBM.

## Introduction

Glioblastoma (GBM) represents the most prevalent and lethal tumor of the central nervous system, constituting 80% of malignant brain tumors^1^. Surgery followed by radiotherapy and TMZ chemotherapy were still the main treatment for primary GBM^2^. With a better understanding of the molecular biology of GBM, several novel therapies targeting the signal transduction pathways, microenvironment, and distinct metabolism were developed^3–5^. However, none of these treatments has been shown to improve the overall survival of newly diagnosed patients with GBM, and the prognosis is still frustrating. Some low-grade gliomas would progress to high-grade gliomas in 7–10 months after the first surgery. Alarmingly, the median survival for patients with GBM was only 14.6 months^6^. Hence, there is an urgent need to identify novel molecular targets and biomarkers to develop efficient therapeutic strategies for GBM.

Mitochondria is a double-membrane-bound organelle that has a distinct structure and unique genome. As bioenergetic and biosynthetic factories, mitochondria are critical for normal cell function and human health^7–9^. Besides, mitochondria are also involved in ROS production, protein quality control, regulation of apoptosis, and mitophagy under pathological conditions^7^. Increasing studies have demonstrated that mitochondria play a central and multifunctional role in tumor formation and progression^10,11^. Compared with normal cells, mitochondria in tumor cells are more susceptible to metabolic stimuli and other changes, owing to their difference in structure and function^12^. Furthermore, critical mitochondrial gene mutations and protein dysfunction significantly contribute to gliomagenesis and progression^13–15^. Consequently, mitochondria-related genes (MRGs) hold promise as prognostic biomarkers and therapeutic targets for GBM.

This study screened nine prognostic MRGs of GBM and constructed an MRGs-based Prognostic Model. Leveraging multivariate Cox analysis, the model was validated as an independent risk predictive factor for patients with GBM. Thus, the nine MRGs-based signatures may be considered a potential and robust diagnostic factor for patients with GBM. To confirm the feasibility of the model, we selected SHC1, the only MRG whose role has not been elucidated in GBM yet, for further in vitro study and found its longest isoform p66Shc was upregulated in GBM tissue. Furthermore, we demonstrated that p66Shc knockdown inhibited the proliferation and migration of GBM cells by disturbing mitochondrial ROS production and morphology.

## Materials and methods

### Method

#### Data acquisition

The gene expression profiles of TCGA-GBM (RSEM TPM) and TCGA TARGET GTEx (RSEM TPM) cohorts and clinical information were downloaded from the UCSC Xena browser (https://xenabrowser.net/). In addition, an external independent GBM database, GSE61335, was gained from the easy Gene Expression Omnibus (easyGEO, https://easygeo.cn/).

### Mitochondrial-related genes extraction

We screened out 686 MRGs using the uniport database (https://www.uniprot.org/) (Supplementary Table S1). In subsequent studies, only 256 coding proteins were matched in the TCGA-GBM matrix.

### Survival analysis

Kaplan–Meier survival analysis and the Cox proportional hazard model were used to estimate the prognostic value of MRGs based on the TCGA dataset using R packages (survival and survminer).

### Construction MRGs-associated risk model

We first identify the risky MRGs in the TCGA-GBM cohort by performing a univariate Cox regression algorithm. Next, LASSO-Cox regression analysis was utilized to identify nine prognostic MRGs in TCGA-GBM, then the risk model was constructed by multivariate Cox regression analysis. The risk score for each patients with GBM was estimated by the following formula: risk score = ∑_(i = 1)^n▒〖Coef(X_i )*Exp(X_i)〗, Coef(X_i): coefficient value of each gene, Exp(X_i): expression of each gene (see in Supplementary Table S2).

### Gene expression analysis

For the TCGA TARGET GTEx cohort, gene-level transcription estimates as log2(x + 1) transformed rsem_isoform_tpm. The TCGA TARGET GTEx dataset was used to analyze the expression level of SHC1 (isoform ID: ENST00000448116.7) and its three isoforms (p46Shc ID: ENST00000368449.8, p52Shc ID: ENST00000368450.5, and p66Shc ID: ENST00000368445.9) in normal brain tissues and GBM tissues. In addition, only the samples taken from the cerebellar hemisphere or cortex in GTEx and matched TCGA solid tissue normal were included in the normal brain tissue group.

### SHC-correlated analysis

The expression analysis of SHC1 was carried out by Gene Expression Profiling Interactive Analysis 2 (GEPIA2, http://gepia2.cancer-pku.cn/#index)^16^ and the Chinese Glioma Genome Atlas (CGGA, http://www.cgga.org.cn/) databases^17^. The comprehensive analyses of the SHC1, including single nucleotide variation (SNV) mutation, copy number variate (CNV), gene ontology (GO)/Kyoto Encyclopedia of Genes and Genomes (KEGG)/GSEA-Hallmark enrichment analysis, immune infiltration, clinical association in multiple GBM cohorts (CGGA_301, CGGA_325, CGGA_693, GSE108474, GSE16011, GSE33331, GSE83300, GSE74187, GSE75824, GSE43378, GSE43289, GSE42669, GSE7696, E_TABM_898, GSE61335_GPL19184, GSE72951, E_MTAB_3892) were conducted using the Biomarker Exploration of Solid Tumors (BEST) database (http://61.129.70.138:8080/app_direct/BEST/). BEST is an integrated database comprehensive biomarker exploration on large-scale datasets in solid tumors analysis.

### Nomogram construction

The independent clinical factors were enrolled to construct a nomogram by multivariate Cox regression analysis for prognosis estimation, which included Glioma CpG Island methylator phenotype (G_CIMP) status, Isocitrate Dehydrogenase (IDH) mutation, and risk score. Patients with missing data were excluded from the analysis. 111 patients were included in the multivariate Cox regression analysis. The concordance index (C-index) was constructed and applied to calculate to evaluate the predictive accuracy of the nomogram model.

### Statistical analysis

The R software version 4.0.2 was used to analyze the data mentioned above. Kaplan–Meier (KM) survival analyses were utilized to compare the survival differences between different patients with GBM. The statistical significance was performed by log-rank test. Pearson’s correlation coefficient was calculated to assess the statistical linear correlation between different factors. The t-test (groups = 2, equal variance), Wilcoxon test (groups = 2), and Kruskal–Wallis H-test (groups > 2) were used to compare the differences between different groups. All p < 0.05 data was regarded as statistically significant.

### Cell culture and transfection

The human GBM cell lines (U87, SHG44, U251) and the glial cell line (HEB) were purchased from the Shanghai Life Academy of Sciences Cell Library (Shanghai, China). The U87 cells cultured in MEM supplemented with 10% fetal bovine serum, 1 mM glutamine. U87 cells were transfected with siRNA or negative control for 72 h using Lipofectamine RNA iMAX Transfection Reagent (Thermo). The siRNA sequences were as follows: p66Shc siRNA#1 sense: AUGAGUCUCUGUCAUCGCUTT, anti-sense: AGCGAUGACAGAGACUCAUTT; siRNA#2 sense: GCUGCAUCCCAACGACAAATT, anti-sense: UUUGUCGUUGGGAUGCAGCTT.

The cell was incubated at 37 °C for 72 h in a six-well plate or 20 mm cell imaging dish.

### ROS and mitochondrial membrane potential (MMP) measurement

Mitochondrial Superoxide Indicator (Invitrogen, United States) and MitoTracker™ Green (Invitrogen, United States) were diluted to 100 nM and 2.5 µM, respectively. The cell was incubated with MitoSox Red or Mitotracker Green at 37 °C for 30 min. After washing three times by MEM without phenol red, Zeiss). ROS and MMP were measured by the fluorescence intensity of imaging acquired using the confocal laser scanning microscope (LSM780, Zeiss) and flow cytometer (BD Bioscience). Quantification of MMP and ROS fluorescent intensity was analyzed by ImageJ software.

### Immunohistochemistry

Human GBM tissues and its paratumor tissues were surgically obtained from patients undergoing treatment at the Department of Neurosurgery, Xiangya Hospital. Upon collection, the tissues were immediately fixed in 4% paraformaldehyde for 48 h at 4 °C. Subsequent to dehydration with graded alcohol solutions (100%, 85%, and 75%), the tissues underwent blocking by incubation in 3% bovine serum albumin (BSA) at room temperature for 30 min. The primary SHC1 antibody (Proteintech, Wuhan, China) was diluted in PBS and incubated with the tissue at 4 °C overnight. The secondary antibody was incubated at room temperature for 30 min, followed by DAB color developing solution to reveal the color of antibody staining. Dehydrate the tissue slides through 75%, 85%, and 100% alcohol and mount the slide with neutral gum. Images were acquired using a microscope (Nikon DS-U3).

### Immunofluorescence

U87 cells were cultured on coverslips in the six-well plate for 72 h and stained with 100 nM Mitotrtacker Red at 37 °C for 30 min before being fixed by PFA. The cell was solubilized with 0.1% Triton X-100 for 15 min and blocked by 1% BSA at room temperature for 30 min. The primary antibodies were diluted to the desired solution by 1% BSA and incubated with the cell at 4 °C overnight. The coverslips were washed with 3 changes of 0.05% PBST before incubating with the secondary antibodies and for 1 h at room temperature. The cell nucleus was stained using DAPI Staining Solution (Abcam) for 10 min at room temperature. Samples were imaged on a confocal laser scanning microscope (LSM780, Zeiss).

### Flow cytometry assays

The number of U87 apoptosis was analyzed by flow cytometry. TMZ was incubated with the U87 cell transfected with the p66Shc siRNA for 72 h. U87 without TMZ was used as the negative control. Cells were washed with cold PBS three times before tryptic digestion. 300 µl 1 × Binding buffer was added to resuspend the cell. Annexin V-FITC was added and incubated for 15 min in the darkness. The Propidium Iodide Solution was added five minutes before the flow cytometer analyzed the cells. Data acquired from the flow cytometer was then analyzed by FlowJo.

### Western blotting

The U87 cells were lysed in the SDS sample buffer, and protein concentration was quantified using BCA Protein Assay Kit (Thermo). Proteins were separated by SDS-PAGE and blocking was performed for 1 h with 5% nonfat dry milk in TBST at room temperature. Primary antibodies were diluted in 5% BSA. The SHC1 antibody which can detect three isoforms were used (Sangon, Shanghai, China).PVDF membranes were incubated in the diluted primary antibodies at 4 °C overnight and the secondary antibody for 1 h at room temperature.

### Cell proliferation and migration assays

The proliferation speed of U87 cells was measured by Cell Counting kit-8 (Sigma-Aldrich, Shanghai, China). U87 cells were seeded at a density of 1000 cells per well in 96-well plates. Every 24 h, CCK-8 solution was added (10 μL/well) to all wells and incubated for 2 h at 37 °C, followed by absorbance measurement at 450 nm in 210a microplate reader (Model 680 microplate reader, Bio-Rad Laboratories).

After knocking down p66Shc for 48 h, U87 cells were seeded into the upper chambers at a density of 5.0 × 104 cells in 300 µL of serum-free cell culture medium, while 500 µL of medium containing 20% FBS was added into the lower chambers. The cell migration assay was performed using 24-well transwell chambers (Corning, NY, United States). Statistical computations and Statistical graphs were performed by the GraphPad Prism v9.3.

### Ethics statement

The study was conducted in accordance with the Declaration of Helsinki, and approved by the Ethics Committee of Xiangya Hospital (202103708;2021.10.08). Written informed consent has been obtained from the patients for participation prior to the study and to publish this paper.

## Results

### Identification of differential prognosis-associated MRGs in GBM

**Figure 1:**
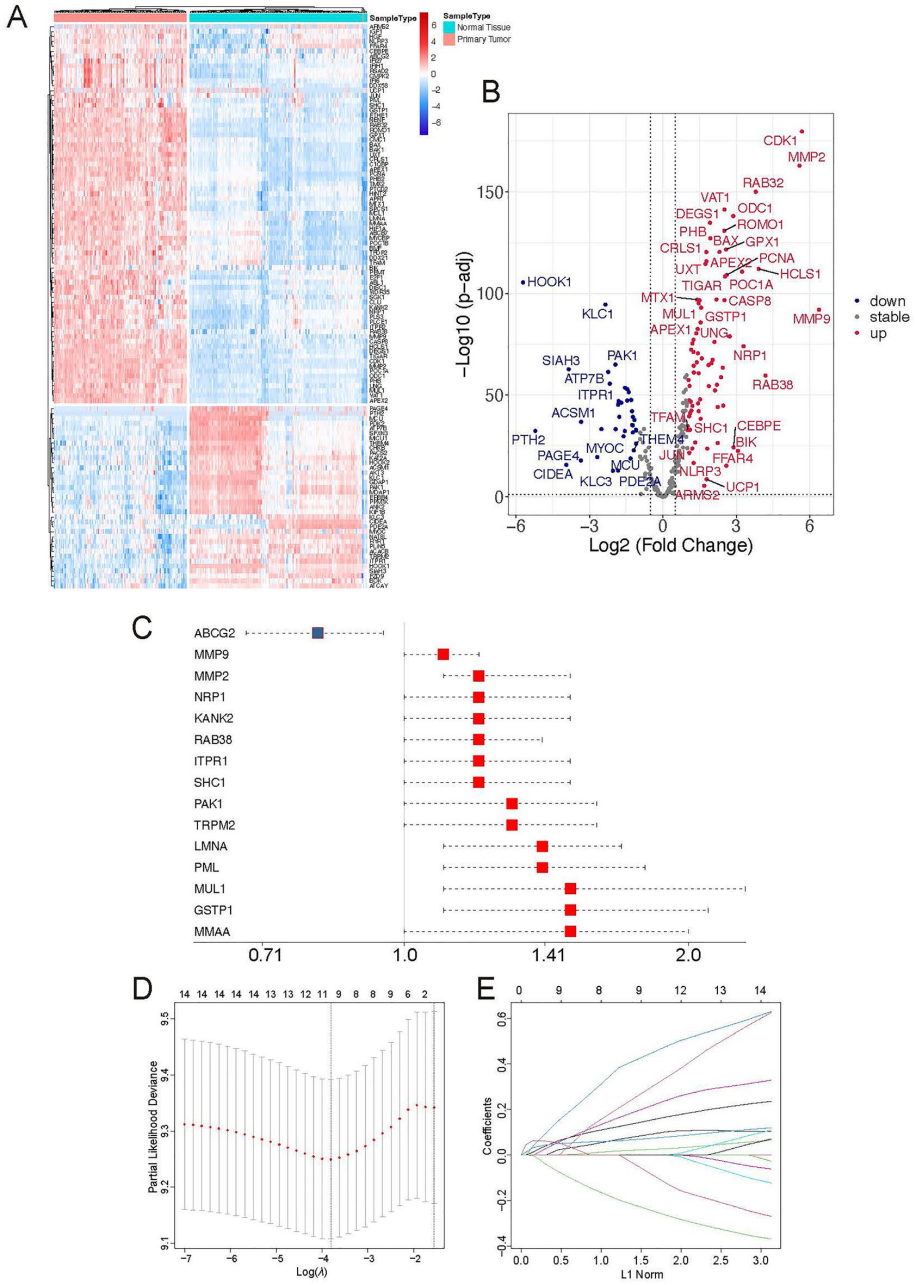
Identification of mitochondrial-related prognostic genes in GBM. (**A**,**B**) The heatmap and volcano plots depict the significantly differential expression of MRGs between primary Glioblastoma multiforme (GBM, n = 152) and paratumor tissues (n = 202). (**C**) Univariate Cox regression analysis examines significantly differential prognosis-associated MRGs. (**D**) LASSO algorithm-associated coefficient profiles of 15 prognostic MRGs. (**E**) The distribution of the optimal lambda value, indicated by the dotted line corresponding to lambda.min.

To evaluate the differential fold change of MRGs in GBM multiforme patients, differential analysis was performed based on the TCGA-GBM dataset. The TCGA cohorts analysis indicated that the expression of 114 genes were significantly different between the GBM tissue and its paratumor tissue, including 77 upregulated genes and 37 downregulated genes (Fig. 1A,B , Supplementary Table S3). Subsequently, univariate Cox analysis was employed to evaluate the prognostic significance of MRGs in patients with GBM based on the TCGA-GBM dataset. The results indicated that 15 genes were associated with the prognosis of GBM. Among the 15 genes, 14 genes [Matrix Metallopeptidase 9 (MMP9), Matrix Metallopeptidase 2 (MMP2), Neuropilin 1 (NRP1), KN Motif And Ankyrin Repeat Domains 2 (KANK2), RAB38, Member RAS Oncogene Family (RAB38), Inositol 1,4,5-Trisphosphate Receptor Type 1 (ITPR1), SHC Adaptor Protein 1 (SHC1), P21 (RAC1) Activated Kinase 1 (PAK1), Transient Receptor Potential Cation Channel Subfamily M Member 2 (TRPM2), Lamin A/C (LMNA), Promyelocytic Leukemia (PML), Mitochondrial Ubiquitin Ligase Activator of NFKB 1 (MUL1), Glutathione S-Transferase Pi 1 (GSTP1) and Methylmalonic Aciduria Type A Protein (MMAA)] had an especially elevated hazard ratio (HR > 1, p-value < 0.05), while only ATP Binding Cassette Subfamily G Membe (ABCG2) had a HR less than 1(p-value < 0.05) (Fig. 1C, Supplementary Table S4). Taken together, these findings suggest that the identified set of 15 MRGs can be utilized to predict the overall survival of patients with GBM within the TCGA cohort.

Although above results demonstrated the predictive efficacy of single MRGs based on the univariate Cox regression analysis, but variables that may affect research outcomes have not been adequately considered. Therefore, the study aimed to develop an integrated prognostic model utilizing these MRGs. To construct this model for assessing the risk profile of each patients with GBM, the least absolute shrinkage and selection operator (LASSO) penalized Cox regression analysis was applied to identify potential prognostic genes. Initially, we evaluated the coefficient values at different levels of penalty (Fig. 1D). Subsequently, we confirmed the optimal lambda value based on the ten-fold cross-validation method (Fig. 1E).

### Construction and validation of the 9 MRGs-based prognostic model

**Figure 2:**
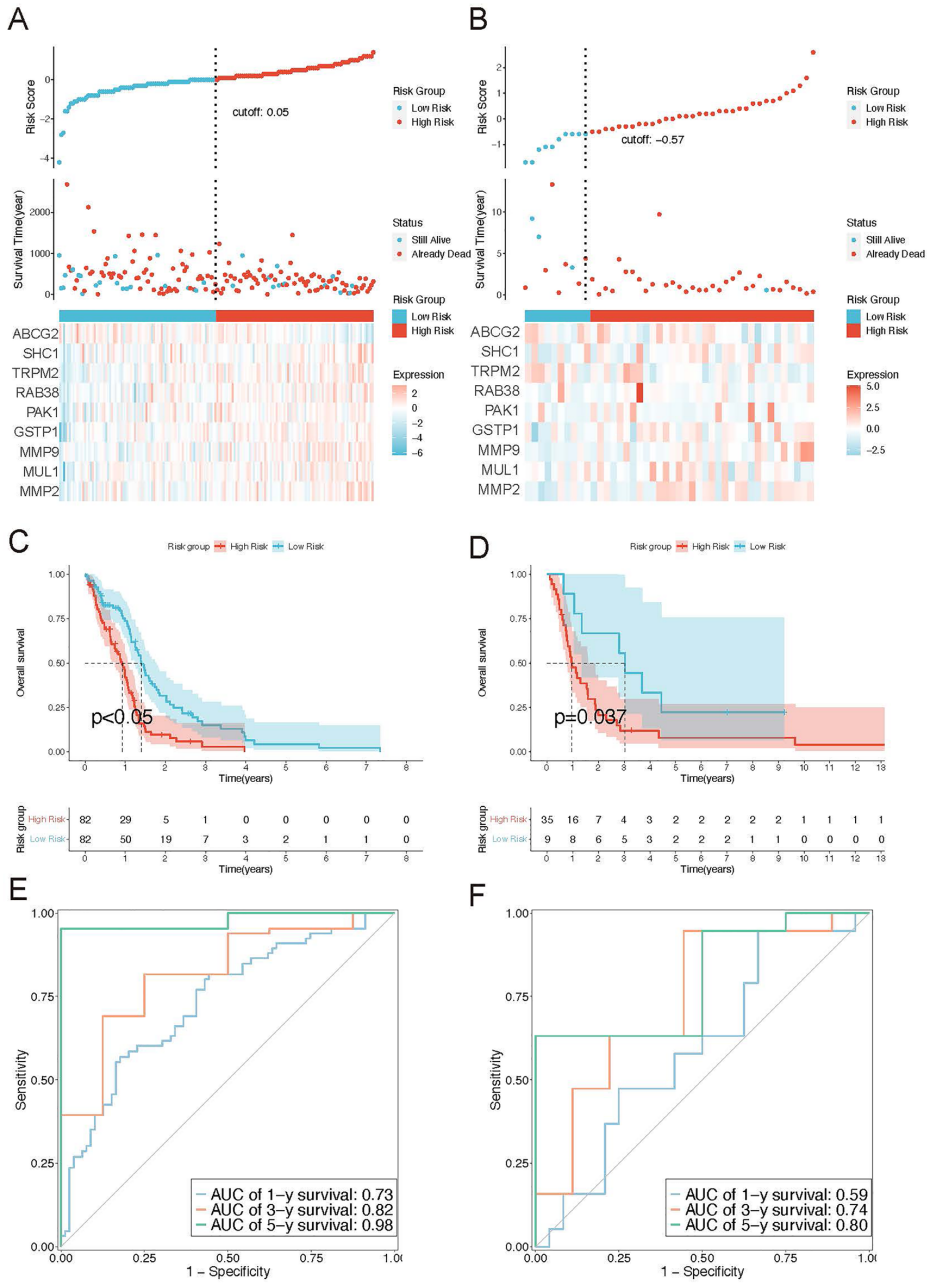
Construction and validation of mrgs risk model. (**A**,**B**) The distribution of each sample of risk group and transcriptome features between the high-risk group and low-risk group in TCGA-GBM (**A**) and GSE61335 (**B**). (**C**,**D**) The overall survival (OS) curves illustrate the differences between high-risk and low-risk groups in TCGA-GBM (**C**) and GSE61335 (**D**). (**E**,**F**) The ROC area under curves of 1, 3, and 5 years in TCGA-GBM (**E**) and GSE61335 (**F**).

The LASSO-Cox regression analysis was employed to evaluate the risk profile of each patient with GBM by identifying nine survival-related genes for the development of a prognostic risk model. These genes, namely ABCG2, SHC1, TRPM2, RAB38, PAK1, GSTP1, MMP9, MUL1, and MMP2, were selected based on their association with survival outcomes. Subsequently, patients with GBM were divided into high- and low-risk group based on the best cut-off value. The distribution of risk scores, patients' statuses, and gene expression patterns between these risk groups in both the TCGA-GBM dataset and GSE61335 is illustrated in Fig. 2A,B. Consistently, Kaplan–Meier survival analysis curves demonstrated a favorable prognosis associated with a low-risk score in both cohorts (Fig. 2C,D). The predictive performance of the risk score for overall survival (OS) was measured by time-dependent receiver operating characteristic (ROC) analysis. As depicted in the area under the curve (AUC), the 9 MRG signature showed robust prognostic validity, with the AUC reaching 0.73 at 1 year, 0.82 at 3 years, and 0.98 at 5 years in the TCGA-GBM cohort. Similarly, in the GSE61335 cohort, the AUC values were 0.59, 0.74, and 0.80 at 1, 3, and 5 years, respectively, indicating the promising performance of the risk model in predicting OS (Fig. 2E,F).

### Independence of the 9 MRGs-based prognostic model in survival prediction

**Figure 3:**
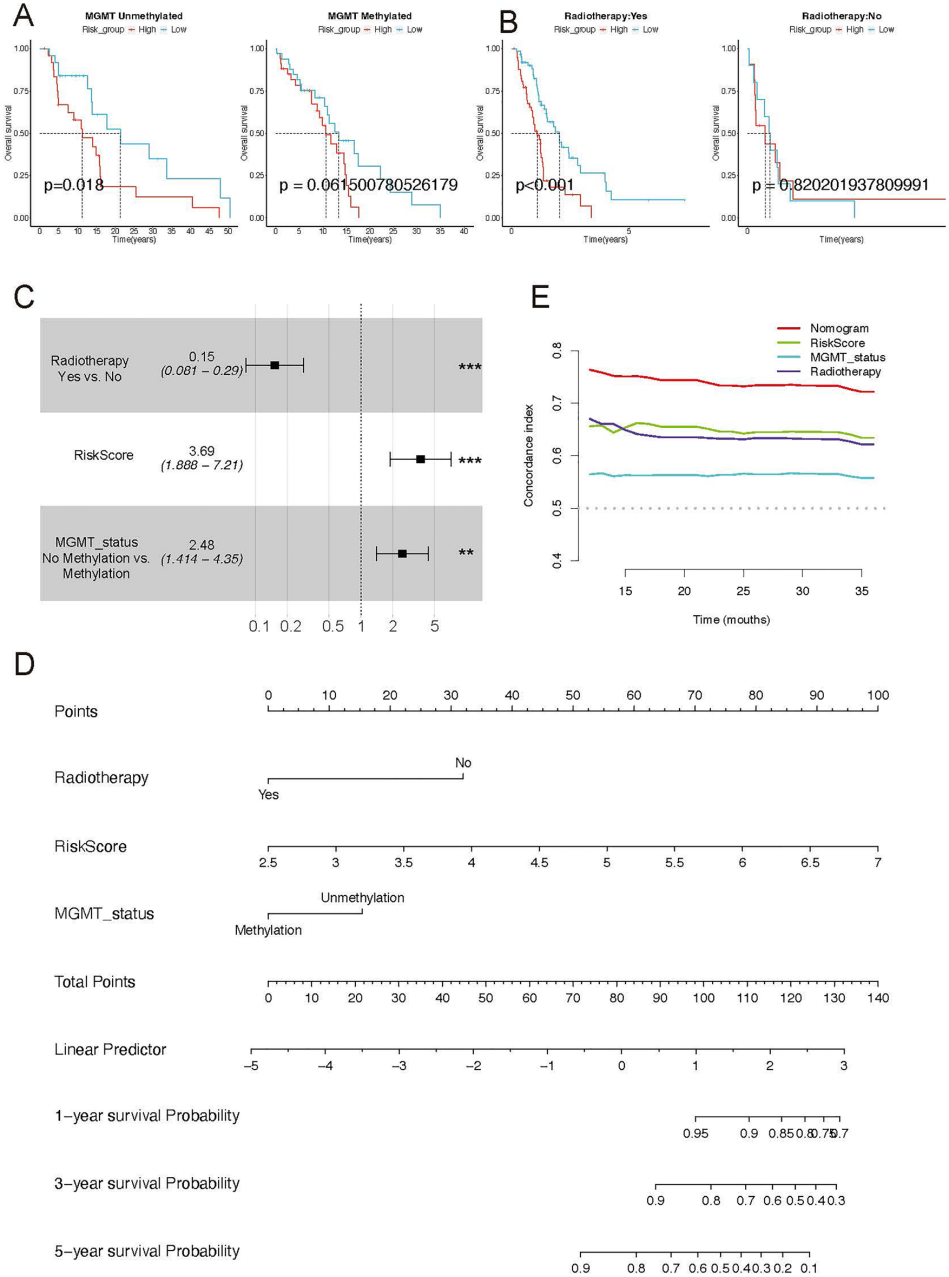
Independent prognostic value of the mrgs signature and construction of the predictive nomogram in the TCGA cohort. (**A**,**B**) The overall survival (OS) differences based on MGMT methylation status (**A**) and radiotherapy (**B**) in the TCGA-GBM dataset. (**C**) The forest plot illustrates two independent clinical prognosis-associated factors and risk scores determined by multivariate Cox regression analysis in the TCGA-GBM cohort. (**D**) A nomogram of the MRGs signature for predicting 1-, 3- and 5-year OS in the TCGA-GBM dataset. (**E**) The time-dependent C-index comparison between the nomogram and other indicators.

We first estimate the subgroup survival variation between the high- and low-risk group. Those GBM patients with MGMT unmethylation, accepted radiotherapy, and IDH wild type are more suitable for performing hazard estimation (Fig. 3A,B, and Supplementary Fig. S1). Multivariate Cox regression analysis of the risk model was performed in the TCGA-GBM cohort to measure the independent predictive ability of the model. Through the multivariate Cox analysis, the risk model was determined to be an independent risk predictive factor for GBM patients (Fig. 3C). After features selection via multivariate Cox analysis (P < 0.05), we established a nomogram model to predict 1, 3, and 5-year survival (Fig. 3D). The concordance index of the nomogram was calculated as the highest value after bias correction and displayed relatively precise performance in clinical diagnosis (Fig. 3E). Thus, these findings suggest that the nine MRGs-based signatures hold potential as robust diagnostic indicators in patients with GBM. Meanwhile, the above results also give us a novel perspective that the abnormal expression of MRGs has played a vital role in the prognosis of GBM.

### SHC1 as a novel prognostic biomarker of GBM

**Figure 4:**
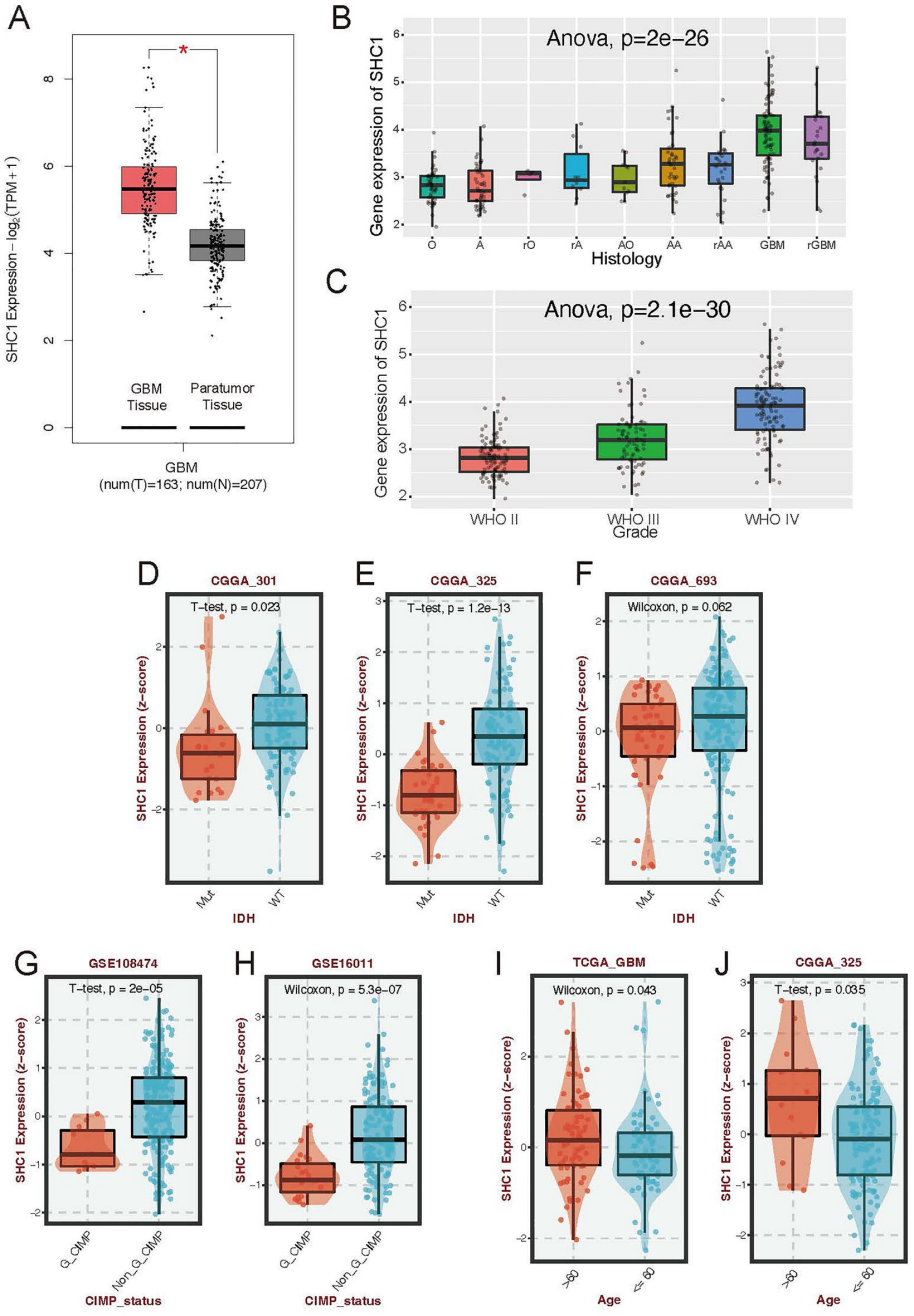
SHC1 is associated with the grade and prognosis of GBM. (**A**) The differential expression of SHC1 between tumor and paratumor tissues of TCGA-Gtex cohort. (**B**,**C**) The distribution of SHC1 expression according to clinical histology stage (**B**) and WHO grade (**C**) in the CGGA_025 cohort. (**D**–**F**) The distribution of SHC1 expression between IDH mutation and wild-type IDH GBM patients in the CGGA database. (**G**,**H**) The distribution of SHC1 expression between G_CIMP status and Non_G_CIMP status patients in GSE108474 (**G**) and GSE16011 (**H**). (**I**,**J**) The distribution of SHC1 expression between patients age more or less than 60 in the TCGA database (**I**) and CGGA_325 (**J**).

Despite demonstrating the independent predictive efficacy of the MRGs model, the feasibility of the model has not been substantiated through in vitro experiments. Consequently, we aim to investigate a novel prognostic MRG in GBM for further analysis. Remarkably, SHC1 emerges as a candidate, as its role in GBM remains unexplored, unlike the other eight MRGs, which have been proved involve in GBM progression^18–24^. Our analysis reveals that SHC1 expression is elevated in GBM compared to paratumor tissue (Fig. 4A). Consistently, the enhanced expression of SHC1 was associated with higher WHO grade of glioma (Fig. 4B,C). To further estimate the relationship between SHC1 expression and clinical subtypes, we analysed the expression of SHC1 in GBM patients with IDH wild-type (WT)/mutant (Mut) and G_CIMP status. Noticeably, patients with GBM without IDH mutation exhibit increased SHC1 expression in CGGA datasets (Fig. 4D–F); whereas G_CIMP positive GBM have lower SHC1 expression in GSE108747 and GSE16011 datasets (Fig. 4G,H). We also found the expression of SHC1 was decreased in GBM patients age 60 or older (Fig. 4I,J).

### Transcriptome differences between SHC1 expression in patients with GBM

**Figure 5:**
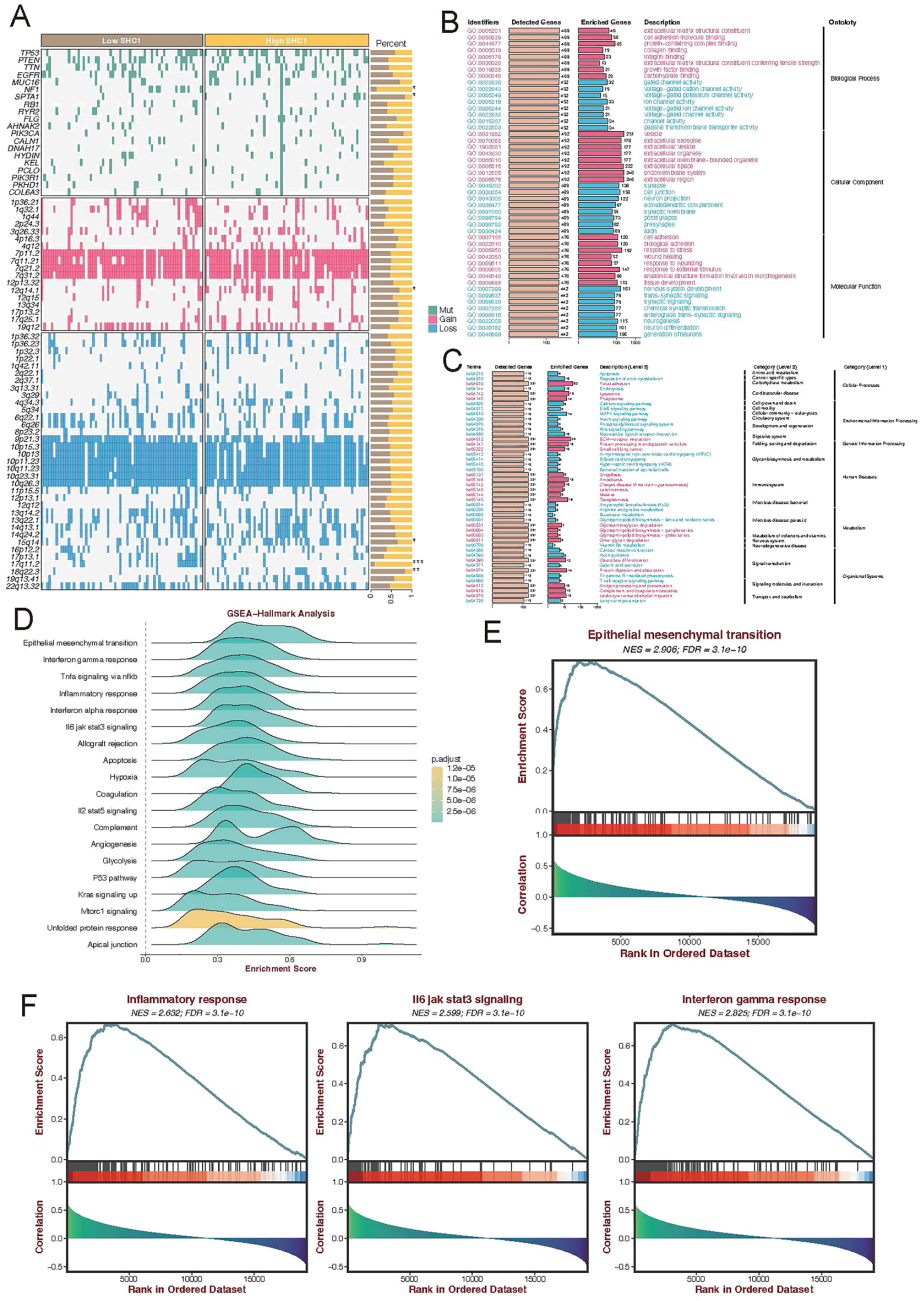
The transcriptional characteristic of SHC1 expressions in GBM. (**A**) Single nucleotide variation and copy number variate mutation between high SHC1 expression and low SHC1 expression patients in TCGA-GBM cohort. (**B**,**C**) Significant linear correlation between SHC1 expression with GO and KEGG pathway enrichment items. Pink front: positive correlation; blue front: negative correlation. (**D**) The ridge plot displays 19 significantly enriched score items of the GSEA-hallmark analysis in the TCGA-GBM cohort. (**E**,**F**) The enrichment rank plot illustrates the positive correlation between SHC1 expression and cancer-associated projects, as well as immune checkpoint blockade therapy-associated signaling pathways.

We performed mutation-associated and enrichment analyses to evaluate the differences between patients with GBM with abnormal SHC1 expression. In patients with GBM, elevated SHC1 expression was associated with single nucleotide variation (SNV) of NF1 and loss of 17p13.1; whereas decreased SHC1 expression was correlated with SPTA1 mutation, loss of 14q24.2 and 18q22.3, and gain of 2q14.1 (Fig. 5A). Gene Ontology (GO) enrichment analyses revealed that these SHC1-associated genes were significantly positively correlated to the extracellular region and response to the stimulus; while SHC1-associated genes inversely correlate with nervous system development and cell junctions (Fig. 5B). Pathway enrichment analyses demonstrated that these SHC1-associated genes were significantly positively correlated to the signaling molecules which interacted with the immune system; SHC1-correlated genes were extraordinarily negatively associated with the cancer-associated signaling pathways and the T cell receptor signaling pathways (Fig. 5C). Consistently, GSEA-Hallmark analysis reveals significantly elevated enrichment scores for immune-associated and cancer-related signaling pathways, including epithelial-mesenchymal transition, Interferon-gamma response, inflammatory response, and IL6/JAK/STAT3 pathways (Fig. 5D–F).

### SHC1 is correlated with immune infiltration in GBM

**Figure 6:**
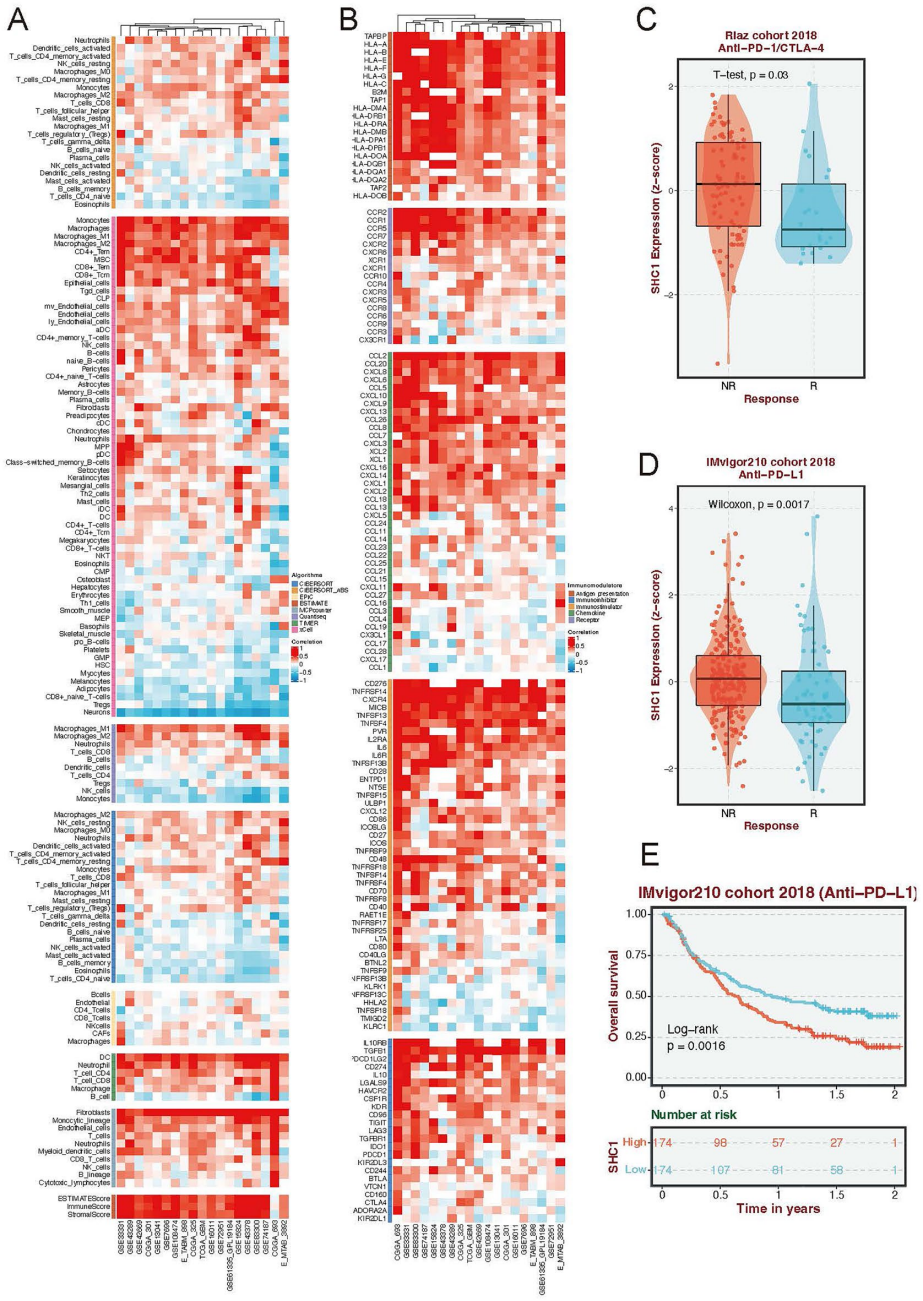
The expression of SHC1 associated with immune cell infiltration in GBM. (**A**) Pearson’s correlation coefficient between SHC1 expression and different immune-associated algorithm scores in multiple GBM datasets. (**B**) Pearson’s correlation coefficient between SHC1 expression and multiple immune-associated genes in different GBM datasets. (**C**) The violet plot displays the differences in SHC1 expression between responder and non-responder treated immune checkpoint blockade inhibitors (PD-1 and CTLA-4) in Riaz cohort 2018. (**D**) The violin plot illustrates differences in SHC1 expression between responders and non-responders treated with PD-L1 in the IMvigor210 cohort 2018. (**E**) Survival curves depict differences between high-SHC1 and low-SHC1 groups in the IMvigor210 cohort 2018.

The enrichment results further explore the correlation between SHC1 expression and immune infiltration in patients with GBM. We conducted eight estimations of immune-associated infiltration to assess the Pearson correlation between the immune microenvironment and SHC1 expression, utilizing algorithms such as CIBERSORT, CIBERSORT_ABS, EPIC, ESTIMATE, MCPcounter, Quantiseq, TIMER, and xCell. Evaluation of SHC1 expression across various GBM datasets (Fig. 6A) revealed a positive correlation with immune infiltrate levels of macrophages, monocytes, neutrophils, dendritic cells, CD4 + T memory cells, and CD8 + T cells. Conversely, SHC1 expression exhibited a negative correlation with Tregs. Furthermore, utilizing the Pearson algorithm, we observed correlations between SHC1 and numerous immune-associated genes in GBM (Fig. 6B), particularly in relation to antigen presentation, immune inhibition, and chemokine receptor activity. Subsequently, we examined differences in SHC1 expression among responder (R) and non-responder (NR) patients receiving immune inhibitor therapy. PD-1/PD-L1 inhibitors are a group of immune checkpoint inhibitors as front-line treatment of multiple types of cancer. Thus, we aim to explore the correlation between SHC1 expression and response to these therapies. In the Riaz cohort 2018, non-responders undergoing PD-L1/CTLA-4 therapies displayed elevated SHC1 expression (Fig. 6C). Similarly, non-responsive patients treated with PD-1 exhibited increased SHC1 expression in the IMvigor210 cohort 2018 (Fig. 6D). Notably, patients with lower SHC1 expression who received PD-1 treatment showed improved survival outcomes (Fig. 6E). These findings suggest that SHC1 may play a significant role in the immune-inflamed environment associated with GBM.

### p66SHC is correlated with the prognosis and survival of patients with GBM

**Figure 7:**
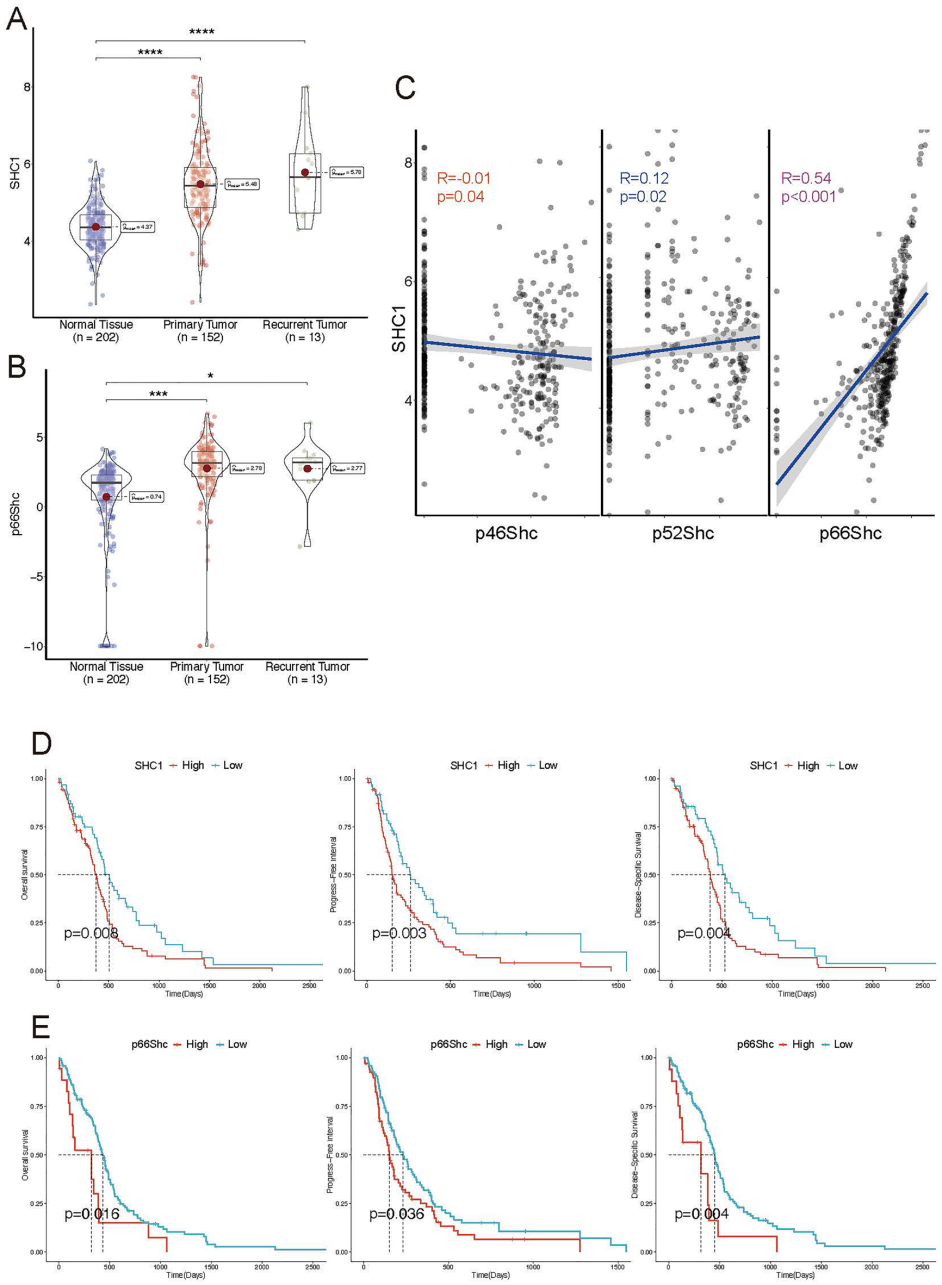
p66SHC is correlated with the prognosis and survival of patients with GBM. (**A**,**B**) The differential SHC1 expression among normal brain tissues, primary tumor, and recurrent tumor. ****p < 0.001, ***p < 0.01, *p < 0.05. (**C**) The linear correlation between SHC1 expression and p46Shc, p52Shc, and p66Shc in TCGA-GBM datasets; R: correlation coefficient, p: p-value. (**D**,**E**) The survival curves for OS, progress-free interval, and disease-specific survival depicted the differences between high and low-SHC1 (**D**)/p66Shc (**E**) groups in the TCGA-GBM cohort.

The human SHC1 gene encodes three proteins, named based on their molecular weight: p46Shc, p52Shc, and p66Shc^25^. We further investigated whether these isoforms are differentially expressed in GBM. Analysis of the TCGA-GBM dataset revealed significant upregulation of SHC1 and p66Shc in GBM compared to paratumor tissue (Fig. 7A and Supplementary Fig. S2A,B). Furthermore, correlation analysis indicated a strong positive correlation between the expression of p66Shc and SHC1, whereas the correlation with other isoforms was weak (Fig. 7B). Additionally, Kaplan–Meier analysis demonstrated that both SHC1 and p66Shc were significantly associated with the prognosis of GBM patients, with high expression predicting shorter overall survival (OS), progression-free interval(PFS), and disease-specific survival (DSS) (Fig. 7C–E), While the expression levels of p46SHC and p52SHC did not correlate with the expression or survival outcomes of patients with GBM (Supplementary Fig. S1C,D). Collectively, SHC1 and p66Shc exhibit significant upregulation in GBM, with p66Shc playing a dominant role in SHC1 expression.Thus,we selected p66SHC for further study.

### p66Shc knockdown inhibits proliferation and migration f U87 cell

**Figure 8:**
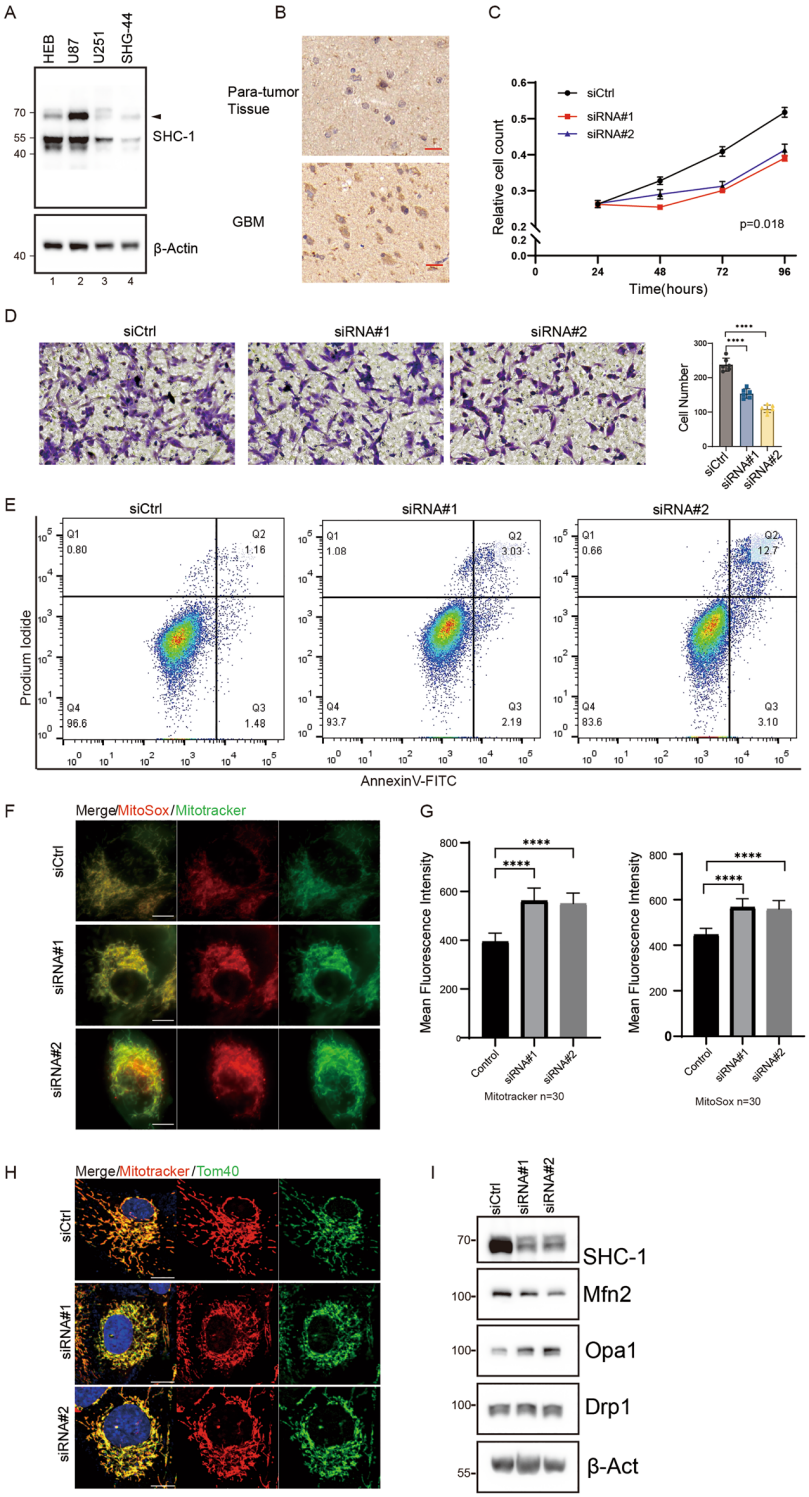
p66Shc promotes proliferation and migration of GBM by mediating mitochondrial functions and morphology. (**A**) Immunohistochemistry detection of p66Shc expression in human GBM samples, showing widespread immunoreactivity in almost all GBM cells (lower panel) compared to only around 10% of cells in adjacent paratumor tissues (upper panel). (**B**) Expression level of p66Shc in different glioma cell lines. (**C**) Cell proliferation in U87 cells transferred with control or p66Shc siRNA was detected by CCK-8 assays. (**D**) Assessment of cell migration ability in U87 cells transfected with control or p66Shc siRNA via Transwell assay. (**E**) Analysis of apoptosis by flow cytometry in U87 cells transfected with control or p66Shc siRNA. (**F**,**G**) Examination of reactive oxygen species (ROS) and mitochondrial membrane potential (MMP) in p66Shc knockdown U87 cells using live-cell microscopy. Quantification of mitochondrial ROS and MMP was conducted by Image J through fluorescence intensity analysis. (**H**) Observations on alterations in mitochondrial morphology following p66Shc treatment, with aggregated mitochondria in p66Shc-treated U87 cells compared to controls. Scale bar = 10 μm. (**I**) Changes in the expression levels of mitochondrial morphology mediator proteins upon p66Shc knockdown in U87 cells.

Since we have found that p66Shc was upregulated in GBM and correlated with the prognosis of the patients with GBM based on bioinformatics analysis. We further confirmed the upregulation of SHC1 in GBM tissues isolated from GBM patients when compared with paratumor tissue (Fig. 8A). To exclude the heterogeneity among tumor cells, we detected the expression level of p66Shc in HEB (human brain normal glial cell) and GBM cells (U87/U251/SHG-44), SDS-page demonstrated p66Shc was only upregulated in U87 cells (Fig. 8B, black arrow). To elucidate the role of p66Shc in GBM, we select U87 for further study and knocked down p66Shc in U87 cells. After incubated with siRNA for 72 h, the proliferation and migration of U87 cells were inhibited (Fig. 8C,D). Flow cytometry assays also indicated that p66Shc knockdown induces apoptosis in U87 cells (Fig. 8E). Altogether, these results suggest that upregulation of p66Shc promotes proliferation and migration of GBM cells and protects them from apoptosis.

### p66Shc regulates mitochondrial functions and morphology in GBM

Elevated basal levels of reactive oxygen species (ROS) in gliomas contribute to high metabolic rates, impacting signal transduction, apoptosis, and the creation of an immunosuppressive environment^26^. To investigate whether apoptosis following p66Shc knockdown resulted from elevated ROS production, MitoSox and Mitotracker were used to study the production of mitochondrial ROS and mitochondrial membrane potential. We observed increased fluorescence intensity of MitoSox and Mitotracker upon p66Shc knockdown in U87 cell, which was also proved by fluoresce analysis (Fig. 8F,G). Furthermore, live-cell fluorescence imaging revealed changes in mitochondrial morphology following siRNA treatment (Fig. 8F). But we can’t elaborate on the change because of the low resolution of the live cell image.

Mitochondrial morphology is regulated by mediators of mitochondrial fusion and fission. DRP1 serves as the key regulator of mitochondrial fission, potentially influencing glioma growth^15^. MFN1, MFN2, and OPA1 are essential for mitochondrial fusion and play pivotal roles in tumorigenesis^27^. Based on the mitochondrial morphology change in the live cell imaging, we further knockdown p66Shc and analyze the expression level of protein related to mitochondrial morphology. After p66Shc knockdown, the mitochondria of U87 cells become aggregated (Fig. 8H). Furthermore, Western blot analysis revealed the downregulation of MFN2 and the upregulation of OPA1 in p66Shc-depleted U87 cells (Fig. 8I). These results indicate the significant role of p66Shc in modulating mitochondrial functions, encompassing both morphology and mitochondrial ROS production.

## Discussion

Mitochondrial dysfunction has been established as a significant feature in malignant gliomas. Notably, mediators of mitochondrial fusion and fission have been implicated in the poor prognosis of GBM, offering potential targets for glioma therapy^15^. Another study demonstrated that the transfer of normal human astrocytic mitochondria into glioma cells enhances aerobic respiration, reduces glycolysis, reactivates mitochondrial apoptosis, suppresses proliferation, and augments the radiosensitivity of glioma radiotherapy^28^. Thus, a comprehensive understanding of Mitochondrial-Related Genes (MRGs) may reveal novel prognostic biomarkers and therapeutic targets for GBM, facilitating the development of effective treatment strategies. In our investigation, we identified 114 differentially expressed MRGs in GBM and developed a novel prognostic model comprising 9 MRGs. The risk score derived from this model showed promising predictive value for the prognosis of GBM patients and exhibited a negative correlation with survival. Subsequent multivariate Cox analysis confirmed that the risk score based on the 9 MRGs was particularly relevant for MGMT unmethylated patients or those who had received radiotherapy. These findings align with previous research indicating that targeting cancer-cell mitochondria and cellular metabolism can enhance the response to radiotherapy^29^.

Among the 9 MRGs comprising the prognostic model, the functions of most genes in the development and progression of GBM have been elucidated^18–24^. For example, For instance, GSTP1 is frequently upregulated in GBM, leading to reduced ROS production, thereby modulating oxidative stress and facilitating GBM cell proliferation^22^. However, the role of SHC1 in the initiation and progression of GBM has not been reported. Based on the TCGA and CGGA database, we found SHC1 was aberrantly upregulated in GBM and its expression was positively correlated with the WHO grade. Furthermore, patients with GBM have lower SHC1 expression levels harbor IDH mutation and G_CIMP, both of which were indicators of long-term survival^30^. The GSEA-Hallmark analysis and GO enrichment analyses identified SHC1 was correlated with multiple pathways involved in cancer proliferation and invasion, including the p53 pathway^31^, angiogenesis^32^, Il6 jak stat3 signaling pathway^33^, and Kras signaling pathway^34^. Moreover, SHC1-associated genes demonstrated positive correlations with immune system signaling molecules. The upregulation of SHC1 activated immune-associated and cancer-related pathways, such as epithelial-mesenchymal transition, interferon-gamma response, inflammatory response, and the IL6/JAK/STAT3 pathway. Our findings corroborate Ahn's discovery that the ShcA pathway triggers STAT3-mediated immunosuppressive signals in breast cancer cells^35^. To elucidate SHC1's role in immune infiltration in GBM, we conducted eight estimations of immune-associated infiltration, revealing a positive association between SHC1 and multiple immune cell infiltrations and adaptive immune processes. Furthermore, elevated SHC1 expression was observed in patients with limited responses to PD-L1/CTLA-4 therapies and PD-1 treatment. These results demonstrate the upregulation of SHC1 in GBM is involvement in immune-associated processes, and its potential as a therapeutic target for immunotherapy.

The human SHC1 gene encodes three distinct isoforms known as p46Shc, p52Shc, and p66Shcc^25^. The p52Shc was reported as a key driver of breast and gastric cancer^36,37^. p46Shc localizes in the mitochondrial matrix, where it hinders thiolase and lipid oxidation processes crucial for tumor metabolism^38^. p66Shc is the longest isoform of SHC1 and consists of 583 amino acids, and 5 functional domains, of which the CH2 domain (proline-rich collagen homology domain) resides in the N-terminal and is critical for pro-oxidant properties^39^. The CCB region (cytochrome C-binding region) within the CH2 domain interacts with cytochrome c and mediates ROS production^40^. Our investigation revealed that among the SHC1 isoforms, only p66Shc exhibited abnormal upregulation in GBM and displayed a correlation with patient prognosis. Thus, we hypothesized that p66Shc is the most critical isoform of SHC1 for GBM development and invasion. Subsequently, we choose to detect the functions of p66Shc in vitro. Following p66Shc knockdown, the proliferation and migration of U87 cells were inhibited. The inhibited proliferation may result from the increased apoptosis after p66Shc knockdown. We also found that knockdown p66Shc increases mitochondrial ROS production in U87 cells, which is consistent with the role of p66Shc in multiple cancers^41–44^. Increased ROS production involved in apoptosis^17^, oncogene expression, and activates of cell signaling cascades, including JAK-STAT signaling pathway progression^45^, inflammatory response^46^, and interferon-gamma response^17^. These findings validated the accuracy of the previous bioinformatic analysis. In addition, Tasseva’s research indicated knocking down p66Shc elevated mammalian mitochondrial ROS production and mediated mitochondrial morphology^47^. We further demonstrated that p66Shc knockdown resulted in the downregulation of MFN2 expression and the upregulation of OPA1 expression in U87 cells, both of which play roles in mitochondria fragmentation. Thus, we propose that the suppression of p66Shc disrupts normal mitochondrial morphology through increased ROS production, subsequently hindering the proliferation and migration of U87 cells.

## Conclusions

In summary, our study constructed a 9 MRGs-based prognostic model which could predict the prognosis of GBM. In this model, we proved SHC1 was upregulated and correlated with the prognosis of patients by involvement in immune infiltration. Moreover, our in vitro experiments elucidated that p66Shc promotes U87 cell proliferation and migration by modulating mitochondrial ROS production and morphology. Therefore, p66Shc emerges as a potential prognostic indicator and promising therapeutic target for GBM.

### Supplementary Information

Supplementary Information 1.

Supplementary Information 2.

Supplementary Information 3.

Supplementary Information 4.

Supplementary Information 5.

Supplementary Information 6.

Supplementary Information 7.

## Data Availability

The datasets used and/or analyzed during the current study are available from the corresponding author on reasonable request.

## References

[1] Ostrom QT, Patil N, Cioffi G, Waite K, Kruchko C, Barnholtz-Sloan JS (2020). CBTRUS statistical report: Primary brain and other central nervous system tumors diagnosed in the United States in 2013–2017. Neuro Oncol..

[2] Stupp R (2005). National Cancer Institute of Canada clinical trials, radiotherapy plus concomitant and adjuvant temozolomide for glioblastoma. N. Engl. J. Med..

[3] Wang Q, Hu B, Hu X, Kim H, Squatrito M, Scarpace L, deCarvalho AC, Lyu S, Li P, Li Y, Barthel F, Cho HJ, Lin YH, Satani N, Martinez-Ledesma E, Zheng S, Chang E, Sauve CG, Olar A, Lan ZD, Finocchiaro G, Phillips JJ, Berger MS, Gabrusiewicz KR, Wang G, Eskilsson E, Hu J, Mikkelsen T, DePinho RA, Muller F, Heimberger AB, Sulman EP, Nam DH, Verhaak RGW (2017). Tumor evolution of glioma-intrinsic gene expression subtypes associates with immunological changes in the microenvironment. Cancer Cell.

[4] Mukherjee P, Augur ZM, Li M, Hill C, Greenwood B, Domin MA, Kondakci G, Narain NR, Kiebish MA, Bronson RT, Arismendi-Morillo G, Chinopoulos C, Seyfried TN (2019). Therapeutic benefit of combining calorie-restricted ketogenic diet and glutamine targeting in late-stage experimental glioblastoma. Commun. Biol..

[5] Molinaro AM, Taylor JW, Wiencke JK, Wrensch MR (2019). Genetic and molecular epidemiology of adult diffuse glioma. Nat. Rev. Neurol..

[6] Omuro AM, Faivre S, Raymond E (2007). Lessons learned in the development of targeted therapy for malignant gliomas. Mol. Cancer Ther..

[7] Pfanner N, Warscheid B, Wiedemann N (2019). Mitochondrial proteins: From biogenesis to functional networks. Nat. Rev. Mol. Cell Biol..

[8] Chacinska A, Koehler CM, Milenkovic D, Lithgow T, Pfanner N (2009). Importing mitochondrial proteins: Machineries and mechanisms. Cell.

[9] Zong WX, Rabinowitz JD, White E (2016). Mitochondria and cancer. Mol. Cell.

[10] Gogvadze V, Orrenius S, Zhivotovsky B (2008). Mitochondria in cancer cells: What is so special about them?. Trends Cell Biol..

[11] Wallace DC (2012). Mitochondria and cancer. Nat. Rev. Cancer.

[12] Bellance N, Lestienne P, Rossignol R (2009). Mitochondria: From bioenergetics to the metabolic regulation of carcinogenesis. Front. Biosci. (Landmark Ed).

[13] Turcan S, Rohle D, Goenka A, Walsh LA, Fang F, Yilmaz E, Campos C, Fabius AW, Lu C, Ward PS, Thompson CB, Kaufman A, Guryanova O, Levine R, Heguy A, Viale A, Morris LG, Huse JT, Mellinghoff IK, Chan TA (2012). IDH1 mutation is sufficient to establish the glioma hypermethylator phenotype. Nature.

[14] Fu L, Dong Q, He J, Wang X, Xing J, Wang E, Qiu X, Li Q (2017). SIRT4 inhibits malignancy progression of NSCLCs, through mitochondrial dynamics mediated by the ERK-Drp1 pathway. Oncogene.

[15] Xie Q, Wu Q, Horbinski CM, Flavahan WA, Yang K, Zhou W, Dombrowski SM, Huang Z, Fang X, Shi Y, Ferguson AN, Kashatus DF, Bao S, Rich JN (2015). Mitochondrial control by DRP1 in brain tumor initiating cells. Nat. Neurosci..

[16] Tang Z, Kang B, Li C, Chen T, Zhang Z (2019). GEPIA2: An enhanced web server for large-scale expression profiling and interactive analysis. Nucleic Acids Res..

[17] Cui Q, Wang JQ, Assaraf YG, Ren L, Gupta P, Wei L, Ashby CR, Yang DH, Chen ZS (2018). Modulating ROS to overcome multidrug resistance in cancer. Drug Resist. Update..

[18] Oberstadt MC, Bien-Moller S, Weitmann K, Herzog S, Hentschel K, Rimmbach C, Vogelgesang S, Balz E, Fink M, Michael H, Zeden JP, Bruckmuller H, Werk AN, Cascorbi I, Hoffmann W, Rosskopf D, Schroeder HW, Kroemer HK (2013). Epigenetic modulation of the drug resistance genes MGMT, ABCB1 and ABCG2 in glioblastoma multiforme. BMC Cancer.

[19] Chinigo G, Castel H, Chever O, Gkika D (2021). TRP channels in brain tumors. Front. Cell Dev. Biol..

[20] Wang H, Jiang C (2013). RAB38 confers a poor prognosis, associated with malignant progression and subtype preference in glioma. Oncol. Rep..

[21] Feng X, Zhang H, Meng L, Song H, Zhou Q, Qu C, Zhao P, Li Q, Zou C, Liu X, Zhang Z (2021). Hypoxia-induced acetylation of PAK1 enhances autophagy and promotes brain tumorigenesis via phosphorylating ATG5. Autophagy.

[22] Lei K, Gu X, Alvarado AG, Du Y, Luo S, Ahn EH, Kang SS, Ji B, Liu X, Mao H, Fu H, Kornblum HI, Jin L, Li H, Ye K (2020). Discovery of a dual inhibitor of NQO1 and GSTP1 for treating glioblastoma. J. Hematol. Oncol..

[23] Han Y, Wu Z, Wu T, Huang Y, Cheng Z, Li X, Sun T, Xie X, Zhou Y, Du Z (2016). Tumor-suppressive function of long noncoding RNA MALAT1 in glioma cells by downregulation of MMP2 and inactivation of ERK/MAPK signaling. Cell Death Dis..

[24] Lin JZ, Lin N (2021). A risk signature of three autophagy-related genes for predicting lower grade glioma survival is associated with tumor immune microenvironment. Genomics.

[25] Migliaccio E, Giorgio M, Mele S, Pelicci G, Reboldi P, Pandolfi PP, Lanfrancone L, Pelicci PG (1999). The p66shc adaptor protein controls oxidative stress response and life span in mammals. Nature.

[26] Salazar-Ramiro A, Ramirez-Ortega D, Perez de la Cruz V, Hernandez-Pedro NY, Gonzalez-Esquivel DF, Sotelo J, Pineda B (2016). Role of redox status in development of glioblastoma. Front. Immunol..

[27] Senft D, Ronai ZA (2016). Regulators of mitochondrial dynamics in cancer. Curr. Opin. Cell Biol..

[28] Sun C, Liu X, Wang B, Wang Z, Liu Y, Di C, Si J, Li H, Wu Q, Xu D, Li J, Li G, Wang Y, Wang F, Zhang H (2019). Endocytosis-mediated mitochondrial transplantation: Transferring normal human astrocytic mitochondria into glioma cells rescues aerobic respiration and enhances radiosensitivity. Theranostics.

[29] McCann E, O'Sullivan J, Marcone S (2021). Targeting cancer-cell mitochondria and metabolism to improve radiotherapy response. Transl. Oncol..

[30] Flavahan WA, Drier Y, Liau BB, Gillespie SM, Venteicher AS, Stemmer-Rachamimov AO, Suva ML, Bernstein BE (2016). Insulator dysfunction and oncogene activation in IDH mutant gliomas. Nature.

[31] Le Rhun E, Preusser M, Roth P, Reardon DA, van den Bent M, Wen P, Reifenberger G, Weller M (2019). Molecular targeted therapy of glioblastoma. Cancer Treat. Rev..

[32] Das S, Marsden PA (2013). Angiogenesis in glioblastoma. N. Engl. J. Med..

[33] Johnson DE, O'Keefe RA, Grandis JR (2018). Targeting the IL-6/JAK/STAT3 signalling axis in cancer. Nat. Rev. Clin. Oncol..

[34] Di Nicolantonio F, Arena S, Tabernero J, Grosso S, Molinari F, Macarulla T, Russo M, Cancelliere C, Zecchin D, Mazzucchelli L, Sasazuki T, Shirasawa S, Geuna M, Frattini M, Baselga J, Gallicchio M, Biffo S, Bardelli A (2010). Deregulation of the PI3K and KRAS signaling pathways in human cancer cells determines their response to everolimus. J. Clin. Investig..

[35] Ahn R, Sabourin V, Bolt AM, Hebert S, Totten S, De Jay N, Festa MC, Young YK, Im YK, Pawson T, Koromilas AE, Muller WJ, Mann KK, Kleinman CL, Ursini-Siegel J (2017). The Shc1 adaptor simultaneously balances Stat1 and Stat3 activity to promote breast cancer immune suppression. Nat. Commun..

[36] Yukimasa S, Masaki T, Yoshida S, Uchida N, Watanabe S, Usuki H, Yoshiji H, Maeta T, Ebara K, Nakatsu T, Kurokohchi K, Kuriyama S (2005). Enhanced expression of p46 Shc in the nucleus and p52 Shc in the cytoplasm of human gastric cancer. Int. J. Oncol..

[37] Wright KD, Miller BS, El-Meanawy S, Tsaih SW, Banerjee A, Geurts AM, Sheinin Y, Sun Y, Kalyanaraman B, Rui H, Flister MJ, Sorokin A (2019). The p52 isoform of SHC1 is a key driver of breast cancer initiation. Breast Cancer Res..

[38] Ventura A, Maccarana M, Raker VA, Pelicci PG (2004). A cryptic targeting signal induces isoform-specific localization of p46Shc to mitochondria. J. Biol. Chem..

[39] Lebiedzinska-Arciszewska M, Oparka M, Vega-Naredo I, Karkucinska-Wieckowska A, Pinton P, Duszynski J, Wieckowski MR (2015). The interplay between p66Shc, reactive oxygen species and cancer cell metabolism. Eur. J. Clin. Investig..

[40] Mir HA, Ali R, Mushtaq U, Khanday FA (2020). Structure-functional implications of longevity protein p66Shc in health and disease. Ageing Res. Rev..

[41] Galimov ER, Sidorenko AS, Tereshkova AV, Pletiushkina O, Cherniak BV, Chumakov PM (2012). P66shc action on resistance of colon carcinoma RKO cells to oxidative stress. Mol. Biol. (Mosk).

[42] Jackson JG, Yoneda T, Clark GM, Yee D (2000). Elevated levels of p66 Shc are found in breast cancer cell lines and primary tumors with high metastatic potential. Clin. Cancer Res..

[43] Muniyan S, Chou YW, Tsai TJ, Thomes P, Veeramani S, Benigno BB, Walker LD, McDonald JF, Khan SA, Lin FF, Lele SM, Lin MF (2015). p66Shc longevity protein regulates the proliferation of human ovarian cancer cells. Mol. Carcinog..

[44] Park YJ, Kim TY, Lee SH, Kim H, Kim SW, Shong M, Yoon YK, Cho BY, Park DJ (2005). p66Shc expression in proliferating thyroid cells is regulated by thyrotropin receptor signaling. Endocrinology.

[45] Li X, Wu C, Chen N, Gu H, Yen A, Cao L, Wang E, Wang L (2016). PI3K/Akt/mTOR signaling pathway and targeted therapy for glioblastoma. Oncotarget.

[46] Mittal M, Siddiqui MR, Tran K, Reddy SP, Malik AB (2014). Reactive oxygen species in inflammation and tissue injury. Antioxid. Redox Signal.

[47] Tasseva G, Bai HD, Davidescu M, Haromy A, Michelakis E, Vance JE (2013). Phosphatidylethanolamine deficiency in Mammalian mitochondria impairs oxidative phosphorylation and alters mitochondrial morphology. J. Biol. Chem..

