# Supplementary figures and images for: The mitochondria-related gene risk mode revealed p66Shc as a prognostic mitochondria-related gene of glioblastoma
Source: Sci Rep. 2024 May 19;14:11418. doi: 10.1038/s41598-024-62083-2 (PMC11102912; doi:10.1038/s41598-024-62083-2)

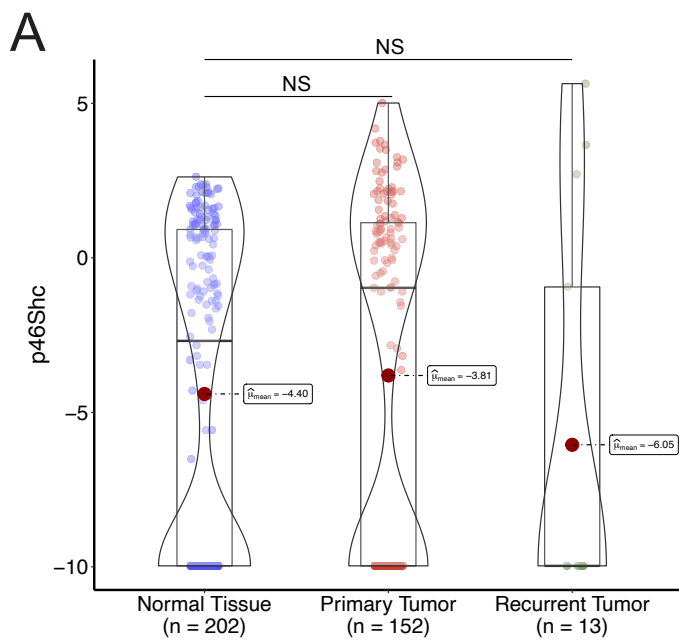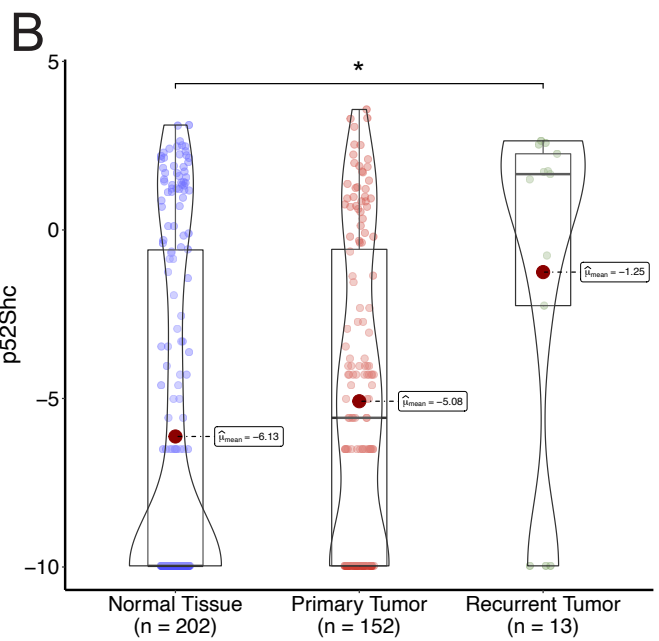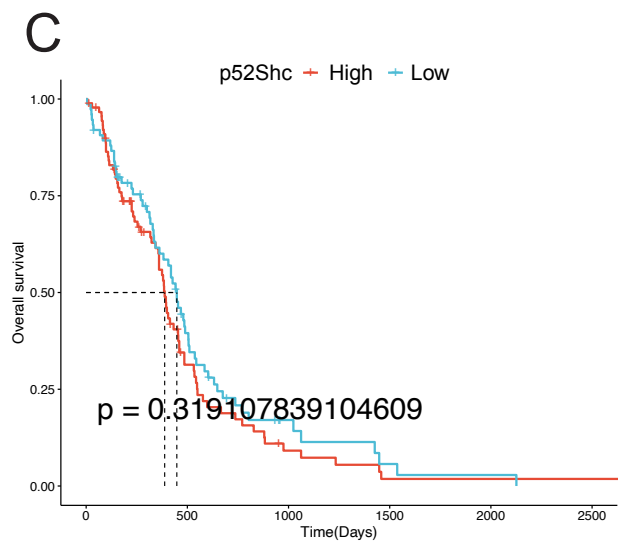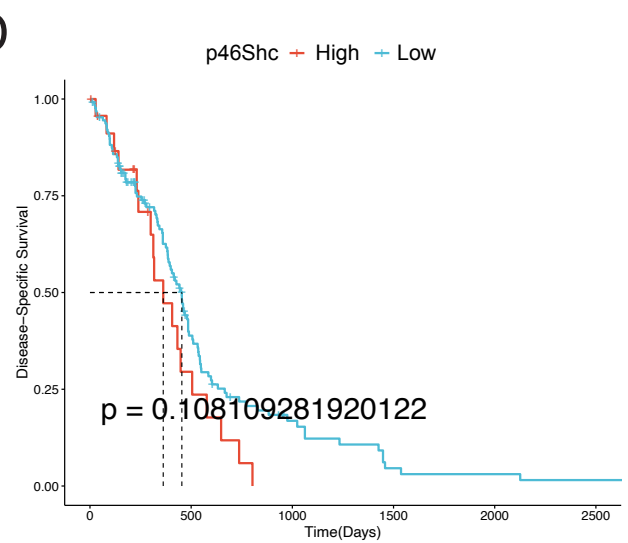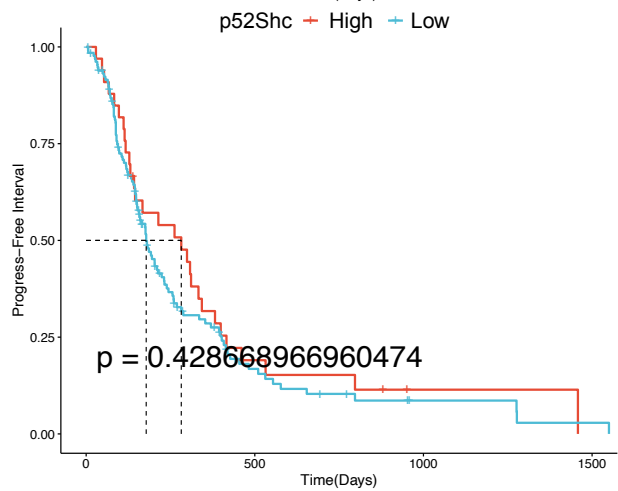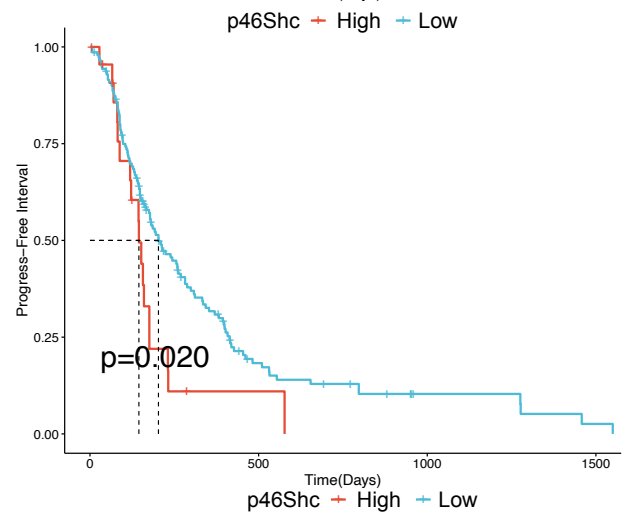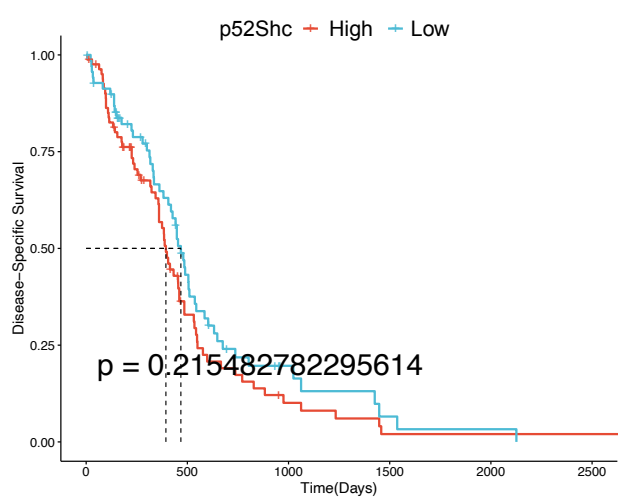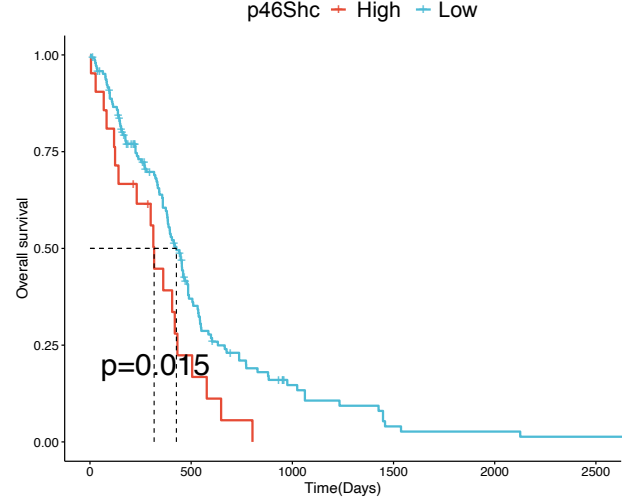

Supplement: Supplementary file 1 — Supplementary Information 1. [file 41598_2024_62083_MOESM1_ESM.pdf]

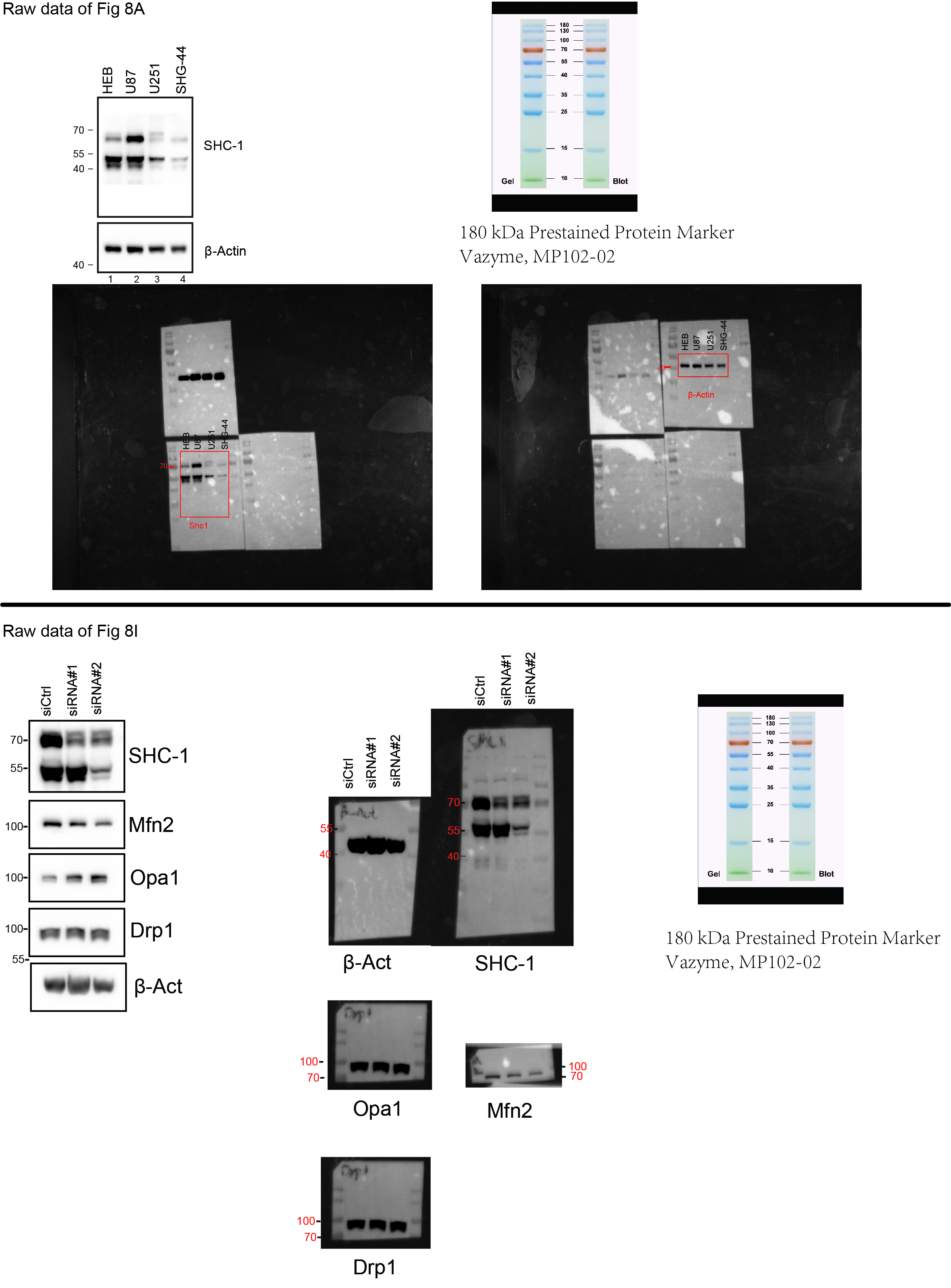

Supplement: Supplementary file 7 — Supplementary Information 7. [file 41598_2024_62083_MOESM7_ESM.jpg]
