# Supplementary material for: The mitochondria-related gene risk mode revealed p66Shc as a prognostic mitochondria-related gene of glioblastoma
Source: Sci Rep. 2024 May 19;14:11418. doi: 10.1038/s41598-024-62083-2 (PMC11102912; doi:10.1038/s41598-024-62083-2)
Supplement: Supplementary file 2 — Supplementary Information 2. [file 41598_2024_62083_MOESM2_ESM.pdf]

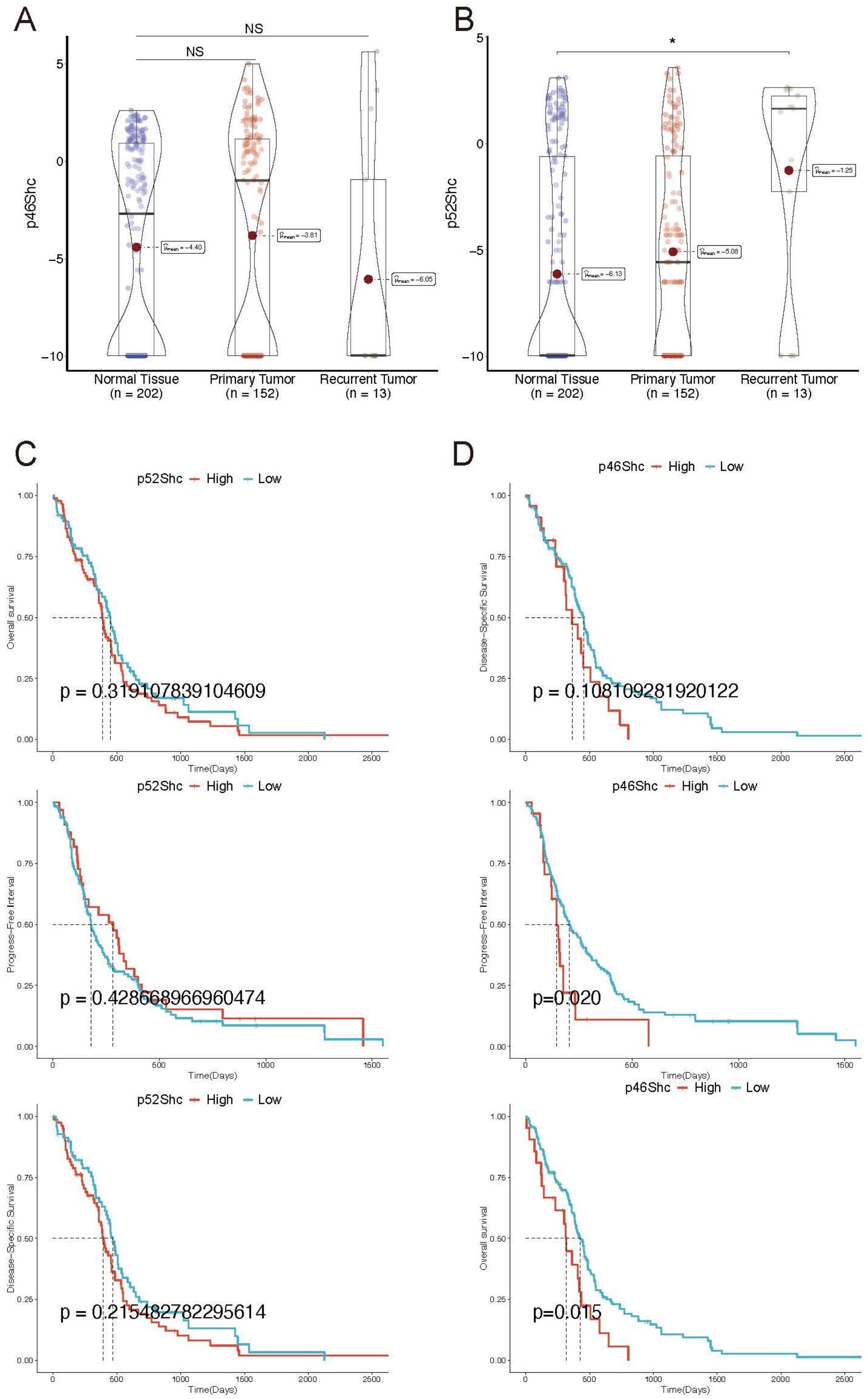

Supplementary Fig. 2 Expression and Survival analysis of p52Shc and p46Shc in GBM. A-B The p52Shc (a) and p46Shc (b) expression among normal brain tissues, primary tumors, and recurrent tumors. **** p<0.001, *** p<0.01, * p<0.05, ns not significant. **C-D** The survival

curves for OS, progress-free interval, and disease-specific survival depicted the differences between high and low- p52Shc (c)/ p46Shc (d) groups in the TCGA-GBM cohort.
